# Nanomechanical thermometry for probing sub-nW thermal transport

**DOI:** 10.1038/s41378-024-00770-w

**Published:** 2024-10-18

**Authors:** Sangmin Oh, Nehpal Singh Shekhawat, Osama Jameel, Amit Lal, Chung Hoon Lee

**Affiliations:** 1 Electrical and Computer Engineering Department, Marquette University, Milwaukee, WI USA; 2 Polytec Inc., 16400 Bake Pkwy, Irvine, CA USA; 3 Electrical and Computer Engineering Department, Cornell University, Ithaca, NY USA

**Keywords:** Sensors, Electrical and electronic engineering

## Abstract

Accurate local temperature measurement at micro and nanoscales requires thermometry with high resolution because of ultra-low thermal transport. Among the various methods for measuring temperature, optical techniques have shown the most precise temperature detection, with resolutions reaching (~10^−9^ K). In this work, we present a nanomechanical device with nano-Kelvin resolution (~10^−9^ K) at room temperature and 1 atm. The device uses a 20 nm thick silicon nitride (SiN) membrane, forming an air chamber as the sensing area. The presented device has a temperature sensing area >1 mm^2^ for micro/nanoscale objects with reduced target placement constraints as the target can be placed anywhere on the >1 mm^2^ sensing area. The temperature resolution of the SiN membrane device is determined by deflection at the center of the membrane. The temperature resolution is inversely proportional to the membrane’s stiffness, as detailed through analysis and measurements of stiffness and noise equivalent temperature (NET) in the pre-stressed SiN membrane. The achievable heat flow resolution of the membrane device is 100 pW, making it suitable for examining thermal transport on micro and nanoscales.

## Introduction

To understand and control phenomena at the micro/nanoscale, one needs to understand thermal transport, which requires high-resolution thermometry^1–4^. The goal of high-resolution thermometry is to resolve the smallest heat flow output by both minimizing the thermal conductance (*G*_th_) and achieving highest temperature resolution (Δ*T*_th_) via ${\dot{Q}}_{{\rm{res}}}={G}_{{\rm{th}}}\times \Delta {T}_{{\rm{res}}}$. Ultra-high-resolution thermometry is required to measure less than nano-watt heat currents for nanoscale heat transport.

Various types of high-resolution temperature sensors, including quantum dots^1^, thermopiles^5^, thermistors^6^, resistance temperature detectors (RTD)^7^, micro-cantilevers^8^, and optical sensors^9^ have been developed for investigating micro/nanoscale thermal transport. In this paper, we present a high-resolution temperature sensor (~10^−9^ K) based on a thin, tensile membrane-based air chamber with a large sensing area > 1 mm^2^.

Due to its light weight, flexibility, flatness, and simple fabrication, a tensile membrane structure has been used in many applications. A few applications of membranes are tabulated in Table 1. Among the materials used for membrane structures, low-stress silicon nitride is preferred due to its robustness and excellent mechanical, chemical, optical, thermal, and electrical properties. A few applications of a low-stress SiN membrane are extreme ultraviolet lithography (EUV) pellicles^10^, infrared (IR) detectors^11^, microphones^12^, transmission electron microscopy (TEM) sample holders^13^. Low-stress SiN membrane has also been used as a platform for micro-calorimeters^14^, all owing to its excellent material properties and its excellent bio-compatible characteristics^13,15,16^. The thickness of the SiN membrane is highly uniform and can easily be controlled at the nanoscale by low-pressure chemical vapor deposition (LPCVD). Furthermore, an LPCVD SiN film can be controlled to have desired tensile/compressive residual stresses and a flat/wrinkle-free membrane. Owing to chemically inert, robust, and bio-compatible characteristics, the SiN membrane allows cells and chemicals to be positioned directly on it. Especially, an ultra-thin (20–25 nm) membrane having an area of 113 mm × 145 mm has been commercially fabricated for EUV pellicles owing to the excellent mechanical properties of the SiN film^10^.

**Table 1:** Applications of membrane structures

Material	Applications	Refs.
SiN	Nanopores	^53^
	Optical absorption	^54^
	Hair flow-sensor	^55^
	Filtration and separation	^56,57^
	EUV pellicles	^10^
	DNA sequencing	^58^
	TEM sample holder	^13^
SiGe	Silicon quantum well	^59^
Al_2_O_3_	Gas sensor	^60^
	Nanopores	^61^
Polymers	Calorimeter	^5^
	Biochemical sensors	^62^
	Microfluidic valve	^63^
	Wearable glucose sensor	^64^

**Fig. 1:**
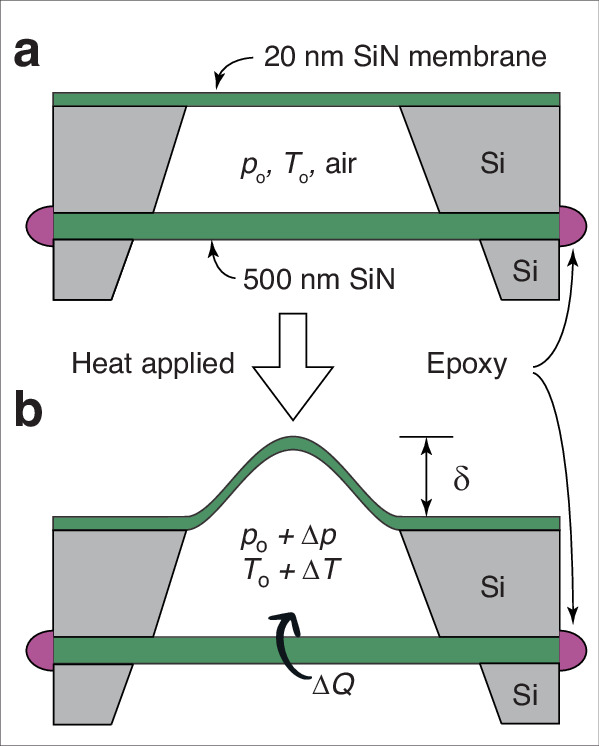
Membrane temperature sensor formation and its principle of operation. The air chamber is sealed by applying epoxy around the devices. **a** An air chamber is formed by the 20 nm thick SiN membrane device and another 500 nm thick SiN film device. **b** Applied heat, Δ*Q*, to the air chamber causes the pressure in the air chamber to increase by Δ*p*. The 20 nm thick membrane deflects by *δ* due to the increased pressure

Nanometers-thick SiN membranes as resonators with a high *Q* factor have been used to detect various physical quantities such as temperature, pressure, and blackbody temperature as well as for photoacoustic imaging^17–22^. The principle of such optomechanical resonators is based on the dependency of the mechanical resonance frequency on temperature due to the thermoelastic effects^17,18^. The resonant frequency shift is correlated to the change in the physical parameters. Numerous optomechanical devices have been developed for sensing temperature change and have achieved up to micro kelvin temperature resolution^17–19^. These optomechanical devices are characterized using an interferometric system in a high vacuum environment and require an external laser to pump the cavity^17,18,21,22^. The detection limit from these devices is quantified by the Allan deviation, intrinsic dissipation in SiN membranes, radiation pressure due to illumination of the membrane with a laser, and the dependency of resonant frequency on the coordinates of the focused laser spot on the membrane^17–21^. This paper proposes using a SiN device working, in principle, off-resonance to measure micro-Kelvin temperature changes. Two SiN membranes with different membrane thicknesses, 20 nm, and 500 nm, are used to create an air chamber. When heat energy is applied to the chamber through the 500 nm SiN membrane, a deflection in the 20 nm SiN membrane is observed due to the change in pressure inside the air chamber, as shown in Fig. 1. The entire surface area of the device forming the air chamber (one side of the 20 nm thick membrane shown in Fig. 1) acts as the sensing area, and micro/nanoscale targets can be placed anywhere on the >1 mm^2^ sensing area, reducing constraints for target placement. The deflection at the center of the 20 nm thick membrane as a function of temperature change can be measured using a monochromatic laser source. While the membrane can be actuated using various methods such as piezoelectric, electromagnetic, and electrostatic, these actuations may interfere with the deflection measurement of the membrane by blocking the optical path of the monochromatic laser source. Instead, the device was thermally modulated to reduce noise. The device’s detection limit is limited by the fundamental thermal actuation of the membrane. The main advantages of nanomechanical membrane device are a large temperature sensing area, easy target placement, and high-temperature resolution (~10^−9^ K), making the device suitable for examining physical, chemical, and biological events at the micro/nanoscale level.

## Device configuration and the principle of operation

**Fig. 2:**
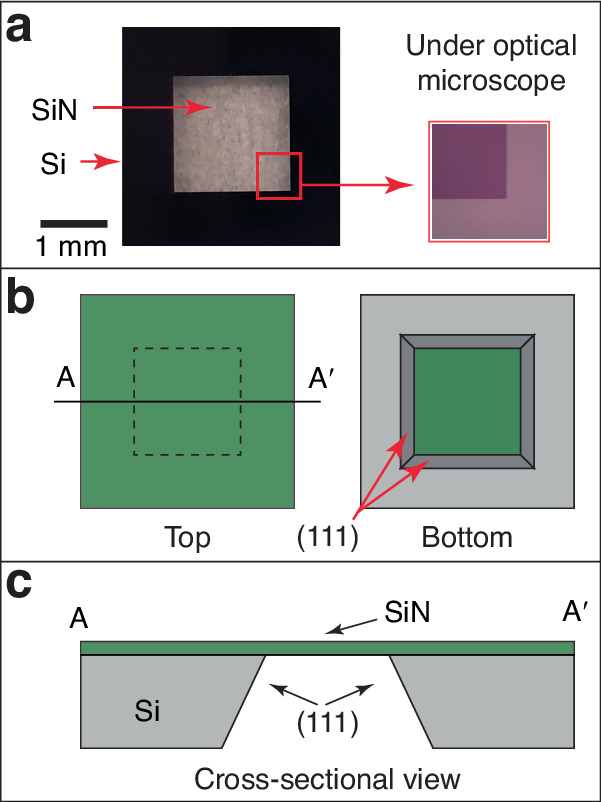
A 20 nm thick SiN membrane device. **a** Overview of the fabricated device. The macroscopic picture shows a transparent SiN membrane, while the optical microscope picture on the right shows the membrane on the left (the red square box shows the membrane). **b** Top and bottom of the device (the thin membrane is green in color). **c** Cross-sectional view, A-A’ in (**b**), of the device. The SiN membrane is suspended by etching Si from the bottom side

The device used in this work consisted of a 20 nm thick SiN membrane and an air chamber. The 20 nm thick SiN membrane was fabricated using cleanroom nanofabrication techniques, including optical lithography, reactive ion etching (RIE), and anisotropic wet silicon etching^14,23^. Figure 2a shows a macroscopic picture of the 20 nm thick SiN membrane on a silicon substrate. Briefly, the membrane was fabricated as follows: a 20 nm thick silicon-nitride (SiN) film was conformally deposited by LPCVD on a 725 *μ*m thick, 8 diameter silicon wafer (resistivity = 10*Ω*-cm) by Rogue Valley Microdevices. Square patterns were patterned on the wafer by optical lithography followed by RIE to selectively remove the SiN film, exposing silicon underneath. The exposed silicon was etched in a potassium hydroxide solution (KOH, 30% w/w) at 60 °C for 28 h. The silicon etch is bounded by the characteristic {111} crystal planes, as shown in Fig. 2b, c. Figure 2c shows the cross-sectional view of the 20 nm thick SiN membrane device. The membrane is suspended flat due to the low tensile residual stress of the SiN film. The residual stress of the SiN film on the 8” silicon substrate after LPCVD is reported by Rogue Valley Microdevices to be a 300 MPa tensile stress at room temperature. The air chamber was formed by placing a 500 nm thick low-stress SiN film device over the etched silicon space and was sealed by epoxy along the device’s edges, as shown in Fig. 1. The 500 nm thick SiN film device was prepared using the same fabrication processes as that used for the 20 nm thick membrane device.

The total in-plane stress (300 MPa) in the film at room temperature, *σ*_t_, is the sum of the stress, *σ*_th_, due to the mismatch of the thermal expansion coefficients of the film and silicon substrate and the intrinsic stress, *σ*_i_: *σ*_t_ = *σ*_th_ + *σ*_i_. The origin of the intrinsic stress is considered to be hindered relaxation between the SiN film and the silicon substrate^24^. However, the intrinsic stress across the film thickness should be balanced out (*σ*_i_ = 0) when the silicon underneath the film is removed. After removing the silicon underneath, the tensile in-plane stress in the suspended SiN membrane turns out to be approximately 11 MPa, as estimated by resonant frequency measurements and ANSYS modal analysis, which are discussed in detail in the following section. The 11 MPa tensile stress in the unsupported SiN film is believed to result from constraints on the film by the silicon surrounding it.

When a small amount of heat, Δ*Q*, is applied to the air chamber through the 500 nm thick film as shown in Fig. 1b, the temperature of the air in the air chamber is increased by,

1 $$\Delta T=\frac{\Delta Q}{n{C}_{v}},$$

where *n* is the number of moles of air in the air chamber, and *C*_*v*_ is the molar heat capacity at constant volume of the air.

From the ideal gas law, the increased pressure, Δ*p*, due to the increased temperature, Δ*T*, in the air chamber can be written as

2 $$\Delta p=\rho {R}_{s}\Delta T,$$

where *p* is the pressure, *ρ* the density of the gas, and *R*_*s*_ the mass specific gas constant. For dry air, *ρ* is 1.225 kg/m^3^ and *R*_*s*_ is 287 J kg^−1^ K^−1^.

From the plate theory, two different approaches can be used to calculate Δ*T* depending on the measured deflection^25^. The total strain energy in a rectangular membrane is the summation of the strain energy from plate bending and the elastic strain energy from internal tensile stresses. When the deflection is smaller than the membrane’s thickness, the majority of strain energy contribution is from the plate bending term, and the plate approach should be applied to calculate the Δ*T*. When the deflection is larger than the membrane’s thickness, the membrane approach should be used. An analysis of the membrane approach is presented for typical applications of the nanomechanical device.

The increased pressure in the air chamber deflects both the 500 nm thick SiN film and the 20 nm thick membranes. The 500 nm thick SiN film is much stiffer than the 20 nm thick membrane; therefore, the 20 nm thick membrane deflects predominantly under the increased pressure in the air chamber. The deflection of the tensile stressed membrane under the applied pressure can be obtained from^25–27^

3 $$\Delta p={C}_{1}\frac{t\sigma }{{a}^{2}}\delta +{C}_{2}(\nu )\frac{tE}{{a}^{4}}{\delta }^{3},$$

where *δ* is the deflection of the membrane at the center, *a* is one-half of the square membrane’s edge length, *t* is its thickness, *E* is its Young’s modulus, *σ* is the stress in the membrane, *ν* is the Poisson’s ratio, and *C*_1_ and *C*_2_(*ν*) are compliance constants specific to the membrane under consideration. In Eq. (3), the contribution from bending (second term) is negligible because the membrane thickness is much smaller than the deflection. For the current membrane, *C*_1_ = 3.04 is used. (In this work, the second term on the right-hand side of Eq. (3) is three orders of magnitude smaller than the first term for typical values of *E* ~ 290 GPa^26^, *σ* from 10 MPa to 300 MPa, *a* ~ 0.5 mm, and *δ* < 100*μ*m)^26^. Therefore, Eq. (3) becomes

4 $$\Delta p(\delta )\approx {C}_{1}\frac{t\sigma }{{a}^{2}}\delta .$$

The membrane stiffness can be determined from Eq. (4) for the present case, in which uniform pressure is applied to the entire membrane surface^28^. For a square membrane the total force, Δ*F*, experienced by the membrane is Δ*F* = Δ*P*(2*a*)^2^ and, thus,

5 $$\Delta F=4{C}_{1}t\sigma \delta ,$$

where *δ* is chosen to be the maximum deflection at the center of the membrane^29^. The resulting stiffness is then determined to be

6 $${k}_{a,d}:=\frac{\Delta F}{\delta }=4{C}_{1}t\sigma ,$$

which for the current membrane gives *k*_*a*,*d*_ = 2.68 N/m. It has previously been shown^25^ that the stiffness, *k*_*a*,*d*_, is approximately four times higher than the stiffness, *k*_*a*,*c*_, obtained by applying a concentrated force at the plate center, i.e., *k*_*a*,*c*_ = *k*_*a*,*d*_/4 = 0.67 N/m for the current membrane. This relationship is verified using atomic force microscopy that applies a concentrated force. The force curve method to obtain the stiffness of the membrane using a commercial AFM and a FEM analysis is presented in the appendix “Stiffness measurement with a concentrated load”.

From Eqs. (2), (4), and (6), the temperature change in the air chamber can be written as

7 $$\Delta T=\frac{{k}_{a,d}}{4\rho {R}_{s}{a}^{2}}\delta .$$

Thus, the temperature change in the air chamber can be determined by measuring *δ*, which can be achieved using various methods such as optical profilometry or a capacitance measurement. Since the dynamic temperature range of the device is within a few degrees Kelvin, any change in parameters such as *ρ* and *R*_*s*_ will be negligible. The temperature resolution is thus limited by the detection sensitivity of the instrumentation and the membrane stiffness.

## Stiffness measurements of a square membrane

Two independent methods were used to verify the above-specified stiffness of the membrane: resonant frequency measurement and ANSYS (FEM) modal analysis. The in-plane stress *σ* is determined by matching the natural resonant frequencies measured using a Polytec MSA-100-3D laser Doppler vibrometer to those obtained by an FEM analysis of a pre-stressed membrane (modal analysis). The same pre-stress (11 MPa) is applied to membranes of different sizes (0.5 mm × 0.5 mm, 1.0 mm × 1.0 mm, and 3.0 mm × 3.0 mm, respectively) because all of the membranes are fabricated as a batch on the same wafer. The thickness, *t*, and the residual in-plane stress, *σ*, are expected to be uniform and vary less than ±5% across the 8” wafer.

### Resonant frequency measurement

The natural frequency of the membrane resonator can be expressed in terms of its stiffness, *k*_*ω*_, and an effective mass (*m*_eff_) as

8 $${\omega }_{o}=2\pi {f}_{1,1}=\sqrt{\frac{{k}_{\omega }}{{m}_{{\rm{eff}}}}}.$$

For a given vibrational mode, the constant of proportionality between the actual membrane mass and its effective mass in Eq. (8) is fixed as the membrane size is varied. This property is used below to verify our claim that the membrane in-plane stress is independent of the membrane size.

In ref. ^30^, a method was presented that enabled the stiffness of the device to be measured from knowledge of the device’s mass and resonance frequency alone. The SiN membrane (1.0 mm × 1.0 mm) is in a highly tension-dominated regime, and, as such, the stiffness comes solely from in-plane stress (11 MPa). This can be shown by conducting a scaling analysis of the plate equation^31^, in which in-plane stresses dominate bending stresses by a factor of 10^7^. Consequently, the method derived (based on ref. ^30^) to measure the stiffness from the frequency correctly accounts for the entire stiffness of the membrane; bending stresses have virtually no contribution.^31^

From ref. ^31^,

9 $$D{\nabla }^{4}\delta \sim {N}_{x}\frac{{\partial }^{2}\delta }{\partial {x}^{2}}+2{N}_{xy}\frac{{\partial }^{2}\delta }{\partial x\partial y}+{N}_{y}\frac{{\partial }^{2}\delta }{\partial {y}^{2}},$$

where *N*_*x*_, *N*_*y*_, and *N*_*xy*_ are the in-plane forces per unit length and *L* = 2*a* is the length of the square membrane. Therefore, the ratio of in-plane stress to the bending stress is

10 $$\frac{N/{L}^{2}}{D/{L}^{4}}=\frac{{L}^{2}\sigma t}{E{t}^{3}/12} \sim 2.2\times 1{0}^{7}.$$

The stiffness for determining the theoretical temperature resolution can be obtained by measuring the *f*_1,1_ frequency. Resonant frequencies (eigenfrequencies) for a stressed square membrane are given by

11 $${f}_{m,n}=\frac{c}{2L}\sqrt{[{m}^{2}+{n}^{2}]},$$

where $c=\sqrt{\sigma /\rho }$ is the speed of sound, and *m* and *n* are positive integers indexing the membrane’s vibrational modes^32^. This is the key result that allows the stiffness to be determined from the measurement of the resonance frequency without knowledge of the stress in the membrane.

From Eq. (11),

12 $$\sigma =\frac{4\rho {L}^{2}}{[{m}^{2}+{n}^{2}]}{f}_{m,n}^{2}.$$

Substituting Eq. (12) into Eq. (6) with *C*_1_ = 3.04 gives an expression for *k*_*ω*_ as a function of *f*_*m*,*n*_,

13 $${k}_{\omega }=\frac{48.64\rho t{L}^{2}}{[{m}^{2}+{n}^{2}]}{f}_{m,n}^{2}.$$

From Eq. (8) and Eq. (13) for *m* = *n* = 1,

14 $${m}_{{\rm{eff}}}/{m}_{o}=0.62,$$

where *m*_*o*_ = *ρtL*^2^ is the mass of the square membrane. For a measured value of *f*_1,1_ = 42.5 kHz for the 1.0 mm × 1.0 mm membrane, Eq. (13) gives *k*_*ω*_ = 2.91 N/m, which agrees with *k*_*a*,*d*_ = 2.68 N/m obtained from Eq. (6). Therefore, *k*_*a*,*d*_ is independently validated by measuring *f*_1,1_.

**Fig. 3:**
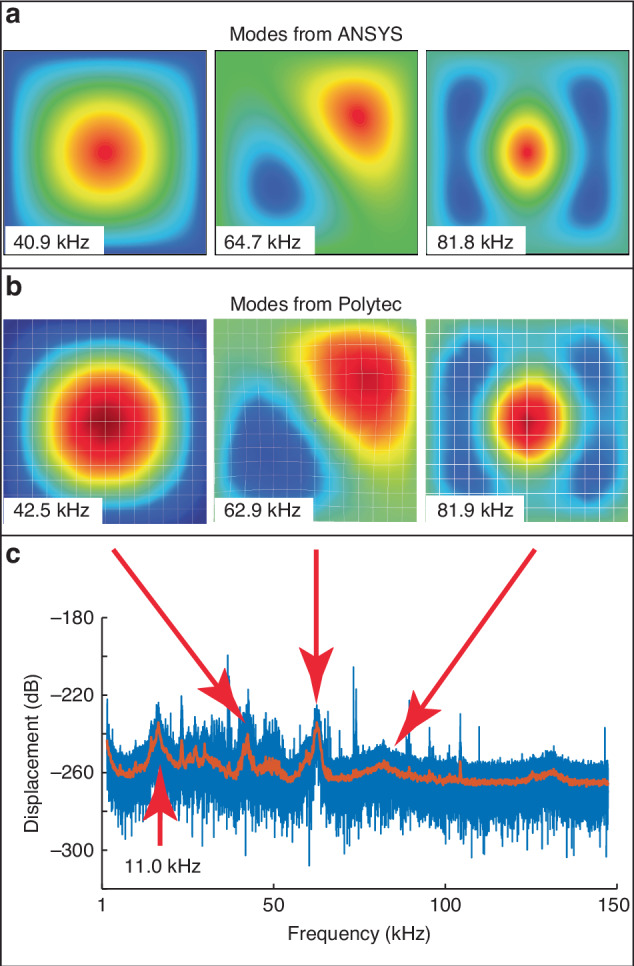
Frequency response of the 1.0 mm × 1.0 mm membrane. **a** Modes (*f*_1,1_, *f*_1,2_, and *f*_1,3_) extracted from ANSYS modal analysis. **b** Measured modes from the Polytec MSA-100-3D. **c** The frequency response of the membrane actuated room temperature thermal noise. The displacement is in dB with respect to 1 m

The resonance spectra and the profiles of the spectral peaks of three different size membranes (0.5 mm × 0.5 mm, 1.0 mm × 1.0 mm, and 3.0 mm × 3.0 mm) are measured by the Polytec MSA-100-3D. The membranes are thermally actuated by room temperature thermal noise (i.e., thermal tuning method). The frequency responses and selective mode shapes of the 1.0 mm × 1.0 mm membrane are shown in Fig. 3. The FEM (ANSYS) modal analysis of the 1.0 mm × 1.0 mm membrane is performed with a pre-stress (residual stress) of 11 MPa applied to the membrane. The modes shown in Fig. 3a are *f*_1,1_ = 40.9 kHz, *f*_1,2_ = 64.7 kHz, and *f*_1,3_ = 81.8 kHz. The modes measured by the Polytec MSA-100-3D (*f*_1,1_ = 42.5 kHz, *f*_1,2_ = 62.9 kHz, and *f*_1,3_ = 81.9 kHz) are shown in Fig. 3b. The Q values of the square membrane for *f*_1,1_, *f*_1,2_, and *f*_1,3_ extracted from a Lorentzian curve fit are ~34, ~59, and ~11, respectively. The measured eigenfrequencies are slightly different from those of the ANSYS modal analysis. This may be due to the uniformity of the residual stress across the membrane and the deviation of the physical membrane size. Figure 3c shows the frequency response of the membrane. There are other resonances below and above the *f*_1,1_. The observed resonances are frame modes, which are non-integer [*m*,*n*] modes.

Those resonances are likely due to external sources or the substrate. For example, the mode at 11 kHz, which is close to the *f*_1,1_ mode at 42.5 kHz, is due to the substrate vibration, as shown in the appendix “Frame modes”. To identify the *f*_1,1_ mode, a well-distinguished mode from frame modes, e.g. *f*_1,2_ and *f*_1,3_ modes of the 1.0 mm × 1.0 mm membrane shown in Fig. 3, are identified from Eq. (15). The frequency of the superposition mode is the same as the single mode. The *f*_1,1_ frequency can be calculated using Eq. (11) and *f*_1,3_, using

15 $${f}_{1,1}=\frac{1}{\sqrt{2.5}}{f}_{1,2}=\frac{1}{\sqrt{5}}{f}_{1,3}.$$

For each membrane size, the measured *f*_1,1_ values, Q, and the stiffness (from Eq. (13)) are summarized in Table 2.

**Table 2:** Membrane size, *f*_1,1_ (kHz) measured by the Polytec MSA-100-3D, and the stiffness (N/m) calculated by Eq. (8) and *m*_eff_/*m*_*o*_ = 0.62

Membrane size	*f*_1,1_	Q	*k*_*ω*_
0.5 mm × 0.5 mm	83.4	47	2.81
1.0 mm × 1.0 mm	42.5	34	2.91
3.0 mm × 3.0 mm	14.6	20	3.10

### Finite element method: modal analysis

A finite element method (ANSYS) is used to calculate the vibrational modes of the membrane for comparison to those obtained by the resonant frequency measurement method. Three modes (*f*_1,1_, *f*_1,2_, and *f*_1,3_) of each membrane are listed in Table 3. A shell element (SHELL281) with quadratic 8-nodes is used for both methods. As boundary conditions, the edges of the membrane are clamped (simply fixed). This shell element is suitable for modeling the bending of a thin membrane. With a solid element, shear blocking can occur. The mechanical properties of SiN film used in the simulations are *E* = 290 GPa, *ρ* = 3300 (Kg/m^3^) and *ν* = 0.28. *σ* is taken to be = 11 MPa.

**Table 3:** Membrane size, *f*_1,1_, *f*_1,2_, and *f*_1,3_ in kHz with *m*_eff_/*m*_*o*_ = 0.62

	ANSYS^a^			Polytec MSA-100 3D		
Membrane size	*f*_1,1_	*f*_1,2_	*f*_1,3_	*f*_1,1_	*f*_1,2_	*f*_1,3_
0.5 mm × 0.5 mm	81.9	129.5	183.1	83.4	127.4	182.0
1.0 mm × 1.0 mm	40.9	64.7	81.8	42.5	62.9	81.9
3.0 mm × 3.0 mm	14.1	22.3	31.5	14.6	23.3	29.9

The mode *f*_2,2_ is lower frequency than mode *f*_1,3_ and is not visible in the Polytec MSA-100 3D measurement. The missing mode *f*_2,2_ due to a high noise floor is also reported in ref. ^65^

^a^Pre-stress (11 MPa) is applied

Since the membrane has residual stress that is directly proportional to the stiffness (Eq. (6)), the pre-stress option (PSTRESS,ON) is applied to the model. Moreover, the pre-stress is applied only in the *x* and *y* directions. The pre-stress (residual in-plane stress) of the silicon nitride is selected to match the *f*_1,1_ frequency measured by the Polytec MSA-100-3D in the section “Resonant frequency measurement”. As large displacement can occur, the “NLGEOM,ON” is activated in the solver. The SiN membrane is considered linear, isotropic, and elastic. A modal analysis is performed to determine the first natural frequency, *f*_1,1_. A few modal solutions (other resonant frequencies) are compared with the measured modes and shown in Fig. 3a. Because the dimensions and material properties of the SiN membrane in the section “Device configuration and the principle of operation” are used, the pre-stress is chosen to match the resonant frequencies measured in the section “Resonant frequency measurement”. For example, the pre-stress determined to match the resonant frequency for the membranes is ~11 MPa.

The stiffness of membranes obtained by ANSYS modal analysis (*k*_A,*ω*_) and the force–distance curve analysis (*k*_A,*F*_ in the appendix “Stiffness measurement with a concentrated load”) is summarized in Table 4. The stiffness value (*k*_A,*ω*_) for each membrane is calculated from Eq. (8) using the *f*_1,1_ frequency from the ANSYS modal analysis and the effective mass, *m*_eff_/*m*_*o*_ = 0.62^33^.

**Table 4:** Membrane size, *k*_A,*ω*_ (N/m) from the ANSYS analysis

Membrane size	*k*_A,*ω*_
0.5 mm × 0.5 mm	2.71
1.0 mm × 1.0 mm	2.70
3.0 mm × 3.0 mm	2.89

## Results

A summary of the stiffness of the 20 nm thick membranes obtained by three methods is given in Table 5. The stiffness of the membrane that is used to calculate the minimum temperature resolution of the device (Eq. (7)). *k*_*a*,*d*_ is analytically calculated from Eq. (6) with a distributed load. *k*_*ω*_ is calculated from Eq. (8) with *m*_eff_/*m*_*o*_ = 0.62 using resonant frequencies measured with the Polytec MSA-100-3D. *k*_*A*,*ω*_ is calculated from ANSYS modal analysis, Eq. (8) with *m*_eff_/*m*_*o*_ = 0.62.

**Table 5:** Membrane size, *k*_*a*,*d*_, *k*_*ω*_, and *k*_A_ in N/m

Membrane size	*k*_*a*,*d*_	*k*_*ω*_	*k*_*A*,*ω*_
0.5 mm × 0.5 mm	2.68	2.81	2.71
1.0 mm × 1.0 mm	2.68	2.91	2.70
3.0 mm × 3.0 mm	2.68	3.10	2.89

### The noise equivalent of temperature

As mentioned briefly in the section “Introduction”, the ultimate heat flow resolution of the membrane device depends on the thermal conductance (*G*_th_) between the thermally isolated region and the thermal reservoir and the resolution (${T}_{{\rm{res}}}$) of the thermometer^34^. The thermal conductance depends on the geometry and configuration of the device. The lumped thermal conductance of the membrane device is measured by a step power response described in the appendix “Device’s thermal parameters” and is ~28 × 10^−3^ W/K at 293 K (room temperature). Once the thermal conductance is measured, the average deflection (*δ*) at the center of the membrane is measured to determine the temperature resolution ($\Delta {T}_{{\rm{Res}}}$) and the resolution of heat flow of the device. Power spectral density (PSD) is measured to obtain *δ*_noise_ and corresponding $\Delta {T}_{{\rm{Res}}}$ of the device at DC and 3 Hz, respectively, and is discussed in this section.

The combined effect of all noise sources is quantified by the measured PSD. The noise sources include Brownian noise, thermal noise, ambient noise, equipment noise, photodetector noise, and Polytec laser noise. Additionally, it accounts for the 260 *μ*Pa radiation pressure exerted by the Polytec laser (Polytec laser diameter and power are ~4 *μ*m and 14 *μ*W, respectively). This is further discussed in the appendix “Radiation pressure from the Polytec laser”. Since Brownian noise is two to three orders of magnitude smaller in comparison to the thermal noise^35^, the ultimate achieved temperature resolution from the membrane deflection is limited by the thermal noise.

The *δ* from thermal noise is calculated according to equipartition^36,37^, where each energy storage mode in thermal equilibrium will have an average energy equal to $\frac{1}{2}{k}_{B}T$, where *k*_*B*_ is Boltzmann’s constant (1.38 × 10^−23^ J/K) and *T* is the absolute temperature. A single mode of energy storage is proportional to the spring potential energy of the membrane, $\frac{1}{2}k \,<\, {\delta }^{2} >$, where *k* is the stiffness of the membrane^38,39^ and *δ* is the deflection of the membrane. With a stiffness of 2.68 N/m for the 20 nm thick membrane used in this work, the thermal noise is on the order of 40 picometer at room temperature (293 K). With *k*_*a*,*d*_ = 2.68 N/m and *δ* = 40 × 10^−12^ m for a 1.0 mm × 1.0 mm and a 3.0 mm × 3.0 mm square membrane, the theoretical temperature resolution is 2.1 × 10^−10^ K and 2.6 × 10^−12^ K, respectively.

#### NET measurement schemes

There are four schemes to measure the noise equivalent temperature (NET). The first scheme is for when the device and temperature are unmodulated (DC). The second is for when the device is modulated, and the temperature is unmodulated. The third scheme is for when the device is unmodulated, and the temperature is modulated. The fourth is for when the device and temperature are modulated. Among these four schemes, DC gives the lowest resolution, and modulation of both device and temperature provides the highest temperature resolution. In this section, we present the DC scheme and power modulated at 3 Hz for comparison.

**Fig. 4:**
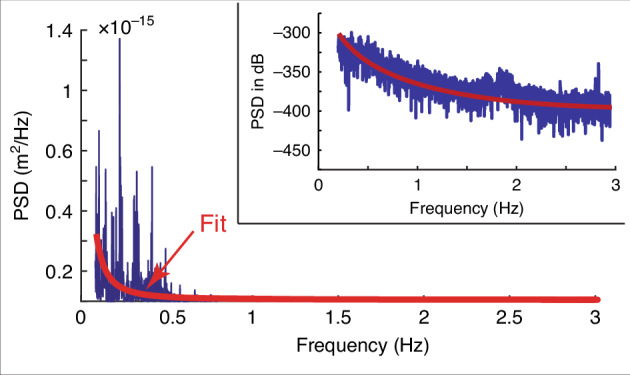
Thermal noise of the 1.0 mm × 1.0 mm membrane. PSD in m^2^/Hz with a fit in red color. The inset shows the PSD in dB with respect to 1 m^2^/Hz

The NET that represents the temperature resolution of the membrane-based thermometry is given by Eq (7). As such

16 $${\rm{NET}}=\Delta {T}_{{\rm{Res}}}={C}_{1}\frac{t\sigma }{\rho {R}_{s}{a}^{2}}{\delta }_{{\rm{noise}}},$$

where the *δ*_noise_ is due to the combined effect of all noise sources. The RMS value of the deflection noise in the bandwidth of interest^40^ can be obtained from

17 $${\delta }_{{\rm{noise}}}={\left[\mathop{\int}\nolimits_{\!\!f}^{f+\Delta f}PS{D}_{{\rm{noise}}}(f)df\right]}^{1/2}$$

where Δ*f* is the bandwidth of measurement. The measured PSD is fitted with *y* = *α* ⋅ *x*^*β*^ + *γ* with coefficients of *α* = 9.02 × 10^−18^, *β* = − 2.43, and *γ* = 2.97 × 10^−17^. The measured PSD of the 1.0 mm × 1.0 mm membrane at room temperature (293 K) in a typical lab environment is shown in Fig. 4. Figure 4 shows that noise is larger at lower frequencies, which is due to the 1/*f* noise arising from thermal and electronic drift in the system.

#### Temperature resolution in DC scheme

For the DC scheme, the *δ*_noise_ is calculated by integrating Eq. (17) with 16 mHz bandwidth for a direct comparison with the results from previous microscale sensor work^41^. The calculated *δ*_noise_ for the DC scheme from Eq. (17) is 2.20 × 10^−9^ m at 293.35 K in a typical open lab environment. *δ*_noise_ in the DC scheme can be lower if the environment is further isolated both thermally and mechanically when measuring the PSD. The calculated NET of the 1.0 mm × 1.0 mm membrane in DC scheme is 1.2 × 10^−8^ K. Previous work^41^ achieved temperature resolution of 10^−3^ K for the DC scheme and 10^−5^ K when both input power and temperature changes were modulated. This device’s achieved DC-temperature resolution exceeds the previous best result achieved in microscale sensor work^41^.

#### Temperature resolution in 3 Hz scheme

The *δ*_noise_ at 3 Hz with the same bandwidth (16 mHz) was calculated using Eq. (17). Modulation improved the temperature resolution achieved in the DC scheme by two orders to 2.3 × 10^−10^ K. The NET achieved in the modulated input power scheme is on the same order as the theoretical temperature resolution limit due to the thermal noise calculated in the section “The noise equivalent of temperature”. The measured *δ*_noise_ and the calculated temperature resolutions for both the schemes are summarized in Table 6.

**Table 6:** Measured *δ* and corresponding Δ*T*

	*δ* (m)	Δ*T*(*K*)
Thermal noise	0.40 × 10^−10^	2.1 × 10^−10^
NET (DC) from measured PSD	2.20 × 10^−9^	1.2 × 10^−8^
NET (3 Hz) from measured PSD	4.32 × 10^−11^	2.3 × 10^−10^
Applied heat power (3 Hz)	7.42 × 10^−10^	5.6 × 10^−9^

16 mHz bandwidth was used for both NET schemes

### Displacement of the membrane as a function of power

**Fig. 5:**
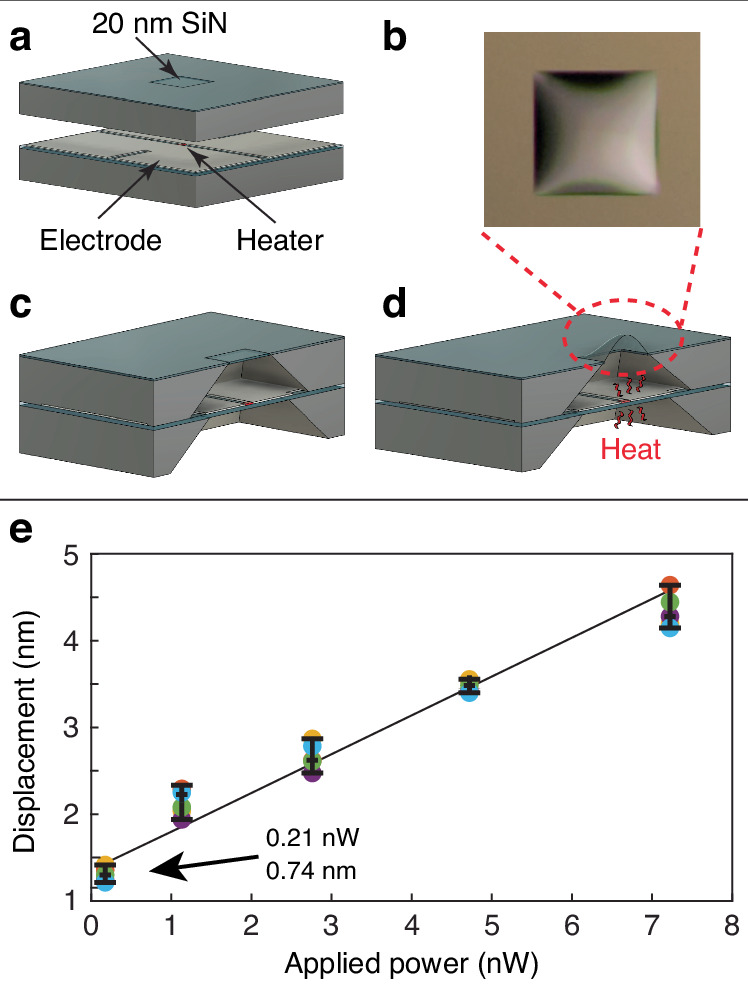
Measured membrane displacement as a function of applied power from a micro-heater. **a** Overview of the 20 nm thick membrane device with a micro-heater device. **b** An optical image of the deflected membrane when an applied heat power from the micro-heater is applied. **c** Cross-sectional view of the device shown in (**a**). **d** Depiction of membrane deflection when Joule heating is applied to the air chamber. **e** Membrane displacement as a function of applied Joule heating power. The average deflection at the center of the membrane is 0.74 nm at 0.21 nW applied power, while the noise floor is ~370 pm

The membrane displacement was measured as a function of applied heat power from a microfabricated heater. The heater was integrated into the device to apply known electrical heat for device characterization. The 20 nm thick membrane device was placed on top of the micro-resistance heater, creating an air chamber that separated the 20 nm thick membrane from the 500 nm thick film. The micro-resistance heater generates sub-nano-watt power as a point source, as shown in Fig. 5. The micro-heater had a conventional serpentine shape (100 *μ*m × 100 *μ*m) with a 5 *μ*m wide and 80 nm thick nickel film on a suspended 500 nm thick SiN film. The 20 nm thick membrane device placement for this measurement is shown in Fig. 5a–d. A Keithley 6221 DC & AC current source and a Keithley 2400 were connected to the micro-heater in a 4-wire measurement configuration. A bias current (*i*_Bias_) was applied to the heater, and the voltage drop across the heater was measured simultaneously. Joule heating from the heater due to the bias current could be calculated as *j*_Power_ = *i*_Bias_ ⋅ *V*_heater_ [W]. The bias current, *i*_Bias_, consisted of two parts: DC bias (*I*_DC_ = 1.5 mA) and AC bias. The DC bias is to maintain steady-state heating. The AC bias serves to apply a minute oscillating power to the air chamber of the membrane device. The frequency of the AC bias was set to 3 Hz, and the deflection of the membrane at the center was measured using the Polytec MSA-100-3D. The deflection at the center of the membrane and the 3 Hz modulated power are shown in Fig. 5e. As shown in Fig. 5e, the smallest measured deflection at the center of the membrane was 0.74 nm at 0.21 nW applied power, while the noise floor was ~370 pm; the signal to noise ratio (SNR) was >2 demonstrating that this device can resolve sub-nano-Watt power. The calculated Δ*T* at 0.21 nW was 5.6 × 10^−9^ K. The addition of Keithley 6221 as a current source increased the electronic noise of the system, and maybe this increase in overall noise can explain the one-order difference between the 3 Hz NET result and the modulated applied heat power result. The results were obtained from repeated deflection measurements performed over several months. Table 6 summarizes the *δ* obtained from the PSD and applied thermal power and their corresponding Δ*T*.

## Discussion

Two applications for studying micro/nanoscale thermal transport are (i) the detection of subtle temperature variations caused by the metabolic activity of single cells in micro/nanoscale samples^1,5,8^, and (ii) the investigation the chemical interactions within nanoscale materials^42–44^. Ensuring effective thermal contact between the sensor and the sample is a critical challenge in such micro/nanoscale thermal transport studies, making the placement of the sample a critical step. Traditionally, this precise positioning requires the use of a precision micro-manipulator, often accompanied by bulky optical equipment, to accurately align the sample on the sensor’s sensitive area. Our sensor design innovates by making the entire surface area of the device forming the air chamber (the other side of the 20 nm thick membrane shown in Fig. 2) act as the sensing area for such micro/nanoscale thermal transport studies. This innovation significantly simplifies the sample placement process on the large membrane, eliminating the dependency on specialized or cumbersome equipment. The design further capitalizes on gravity when the membrane, doubling as the sensing element, is oriented upwards, ensuring reliable thermal contact between the sample(s) and the sensor.

While the device presented in this paper can be used as an ultra-high resolution temperature sensor for chemical sensors, calorimeters, and biosensors, it can also be used as a bolometer to detect near-infrared (NIR), mid-infrared (MIR), far-infrared (FIR), and Terahertz (THz). Specific IR/THz absorption materials can be used on the sensing element membrane. The specific absorption film (e.g. graphene) absorbs IR/THz and converts it to heat, causing the 20 nm thick membrane to deflect. The absorption film, graphene, can be electrically tuned to shift its Fermi level to increase the sensitivity and selectivity. The response time of the sensor depends on the thermal time constant of the air chamber, which is proportional to the volume of the air chamber. Reducing the space between the thick membrane and thin membranes can reduce the device’s thermal time constant and increase its bandwidth. To achieve the desired bandwidth, the two membrane sides can face each other with a space of desired thickness.

When the device is fabricated at room temperature and 1 atm, the membrane can be either concave or convex due to the pressure (temperature) of the sealed air chamber formed during fabrication being different from the environment when the measurement is performed. Therefore, the air chamber needs to be in equilibrium with the environment, limiting the dynamic range of temperature measurement. A microchannel and valve can be integrated to create an equilibrium between the air chamber and the environment, which adds complexity to the sensor system.

The thermal expansion coefficient differences of the silicon/silicon nitride interface on the membrane may affect the deflection. Once the thermal expansion coefficient of the LPCVD silicon nitride is available, the effect of the thermal expansion coefficients can be studied.

The total lumped thermal conductance is largely dominated by ${G}_{{\rm{SiN}}}$ (see the section “Device’s thermal parameters”). The applied heat from the micro-heater is dissipated through the 500 *μ*m thick SiN film. By reducing the thermal conductivity of the SiN film (by reducing the thickness of the film), the power resolution of the device can be improved. The temperature resolution of the device can be obtained by measuring the thermal conductance (*G*_th_) of the device and using the Fourier heat equation ${\dot{Q}}_{{\rm{res}}}={G}_{{\rm{th}}}\times \Delta {T}_{{\rm{res}}}$, where the ${\dot{Q}}_{{\rm{res}}}$ is the applied power to the device at room temperature and 1 atm. As discussed in the section “Device configuration and the principle of operation”, either the plate or the membrane approach depending on the *δ* should be used. The measured *G*_th_ of the device from the step power response is 28 × 10^−3^ W/K as described in the appendix “Device’s thermal parameters”. From the section “Displacement of the membrane as a function of power”, the smallest heat power that the membrane device can resolve is 0.21 nW and *δ* is 0.74 nm. Since the measured *δ* is smaller than the thickness of the membrane (20 nm), the plate approach is applied to calculate $\Delta {T}_{{\rm{res}}}$. From the plate theory, the temperature resolution from the plate approach is given by Δ*T* = ζ ⋅ (*D* ⋅ *δ*/*ρR*_*s*_*L*^4^), where *ζ* is 1/0.00126, $L = 2a$, *L* is the length of the square membrane, *D* is the flexural rigidity, *ρ* is the density of the gas, and *R*_*s*_ is the mass-specific gas constant^25,27^. Using *δ* = 0.74 nm, the corresponding ${\Delta}$*T* is 5.60 × 10^−9^ K. The *G*_th_ is 37.5 × 10^−3^ W/K, which agrees with the measured *G*_th_ of the device (28 × 10^−3^ W/K).

A sophisticated and high-cost optical method (Polytec MSA-100) is used for measuring the displacement of the 20 nm thick membrane. A capacitance measurement approach simplifies the setup, making it low-cost and portable by compromising the resolution to ~10^−3^ K to ~10^−4^ K. To form a capacitive gap, a thin metal layer (~10 nm) needs to be either evaporated or sputtered onto the 20 nm thick membrane acting as a movable electrode. The additional metal film may affect the membrane’s stiffness, which is directly related to the temperature resolution. Another metal electrode on a substrate has to be placed over the metallic 20 nm thick membrane with an appropriate spacer (~10 *μ*m) on the device shown in Fig. 1. The change in the 20 nm thick membrane’s displacement changes the measured capacitance. Analog Device AD7746 can be used to measure the capacitance change. AD7746 has a resolution down to 4 aF, which corresponds to ~ 4 nm displacement of a 1.0 mm × 1.0 mm membrane with an initial spacing of 100 *μ*m between the electrode plate and the membrane.

## Conclusion

A nanomechanical temperature sensor with ~1 nK temperature resolution is presented. The 20 nm thick membrane is fabricated with standard micromachining techniques (optical lithography and KOH wet chemical silicon etching). The 500 nm thick bottom film in Fig. 1 enclosing the air chamber is the sensing element of our device and can have a macroscale area (>1 mm^2^). Because the sensing area is large, a sample can easily be placed at any location on the membrane, and proper thermal contact between the sensing element and nano/microscale samples can easily be achieved.

The theoretical temperature resolution depends on the stiffness of the 20 nm membrane. Our detailed analysis and measurements delineate the contributions of both the stiffness variations of ultra-thin membranes and the thermal noise resulting from the environment to the temperature regulation of the device. Furthermore, based on the measured PSD, our analysis quantifies the achievable noise equivalent temperature as 1.2 × 10^−8^ K for the DC scheme and 2.3 × 10^−10^ K for the 3 Hz modulated scheme, which is of the same order of temperature resolution limit from thermal noise. The deflection measured at the center of the membrane with the applied heat of 0.21 nW was 0.74 nm. The corresponding temperature resolution was 5.6 × 10^−9^ K. The thermal conductance of the device measured from the step power response was 23 × 10^−3^ W/K and agreed with those obtained from the plate approach (37.5 × 10^−3^ W/K), the membrane approach (61.4 × 10^−3^ W/K), and the equivalent thermal network (25.2 × 10^−3^ W/K).

Our work shows that it is possible to resolve temperature changes of ~ 10^−9^ K with the deflection of ultra-thin membrane-based thermometry. By further minimizing the thermal conductance of the device (cf. ${G}_{{\rm{SiN}}} \sim 1{0}^{-3}$ W/K) and achieving a temperature resolution of ~ 10^−9^ K, a heat-current resolution of < 10^−12^ W can be achieved. The heat flow resolution achieved by the 20 nm thick SiN membrane device is suitable for examining nano/microscale thermal transport at room temperature and 1 atm.

## Figures and Tables

**Fig. 6:**
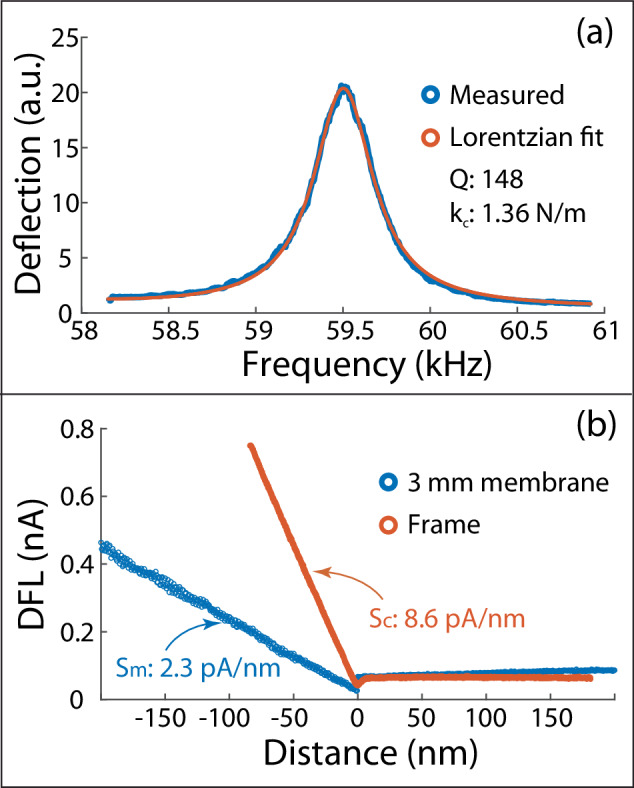
Measurement of the stiffness of the membrane. **a** Thermal tuning of the FCLA-5 probe (Applied NanoStructures, Inc.) and Lorentzian curve fit is used to extract Q and the stiffness of the cantilever. **b** Force curves are measured against the frame and the 1.0 mm × 1.0 mm membrane. The *y*-axis is in nA (cantilever deflection, DFL, signal in photodetector current) instead of the force in N

**Fig. 7:**
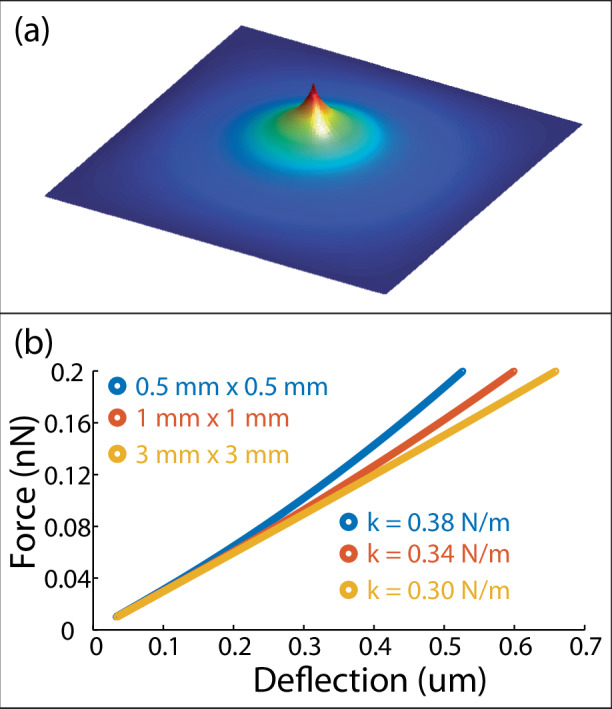
The stiffness of the membranes obtained by ANSYS force–distance curve. **a** Perspective view of the force–distance curve. A known force is applied to a node at the center of the membrane. The displacement of the membrane is scaled (×50) for better visualization of the deformed membrane. **b** Force curves for three different-size membranes. The slopes are larger for smaller membrane sizes

**Fig. 8:**
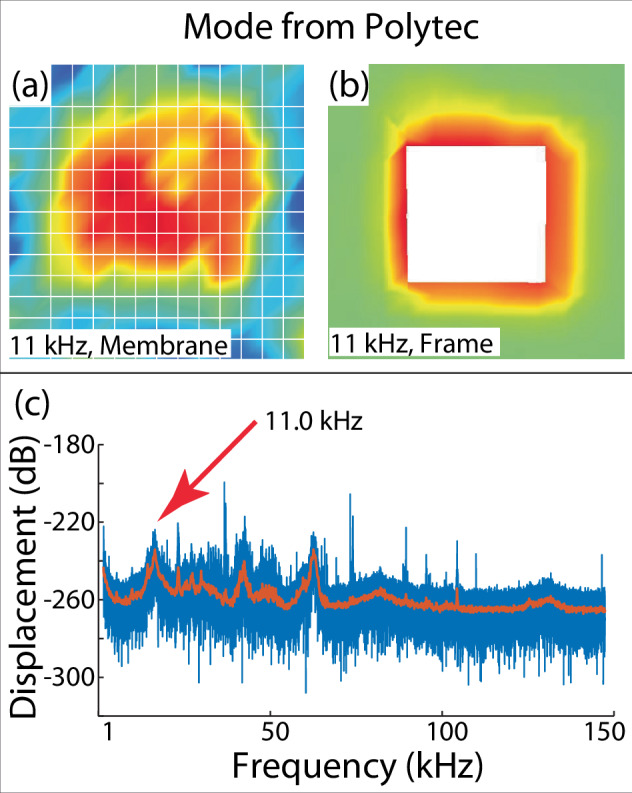
Frequency response of the 1.0 mm × 1.0 mm membrane at 11 KHz. **a** Mode of the membrane. **b** Mode of the frame only (excluding the membrane). **c** The frequency response of the membrane actuated room temperature thermal noise. The displacement is in dB with respect to 1 m

**Fig. 9:**
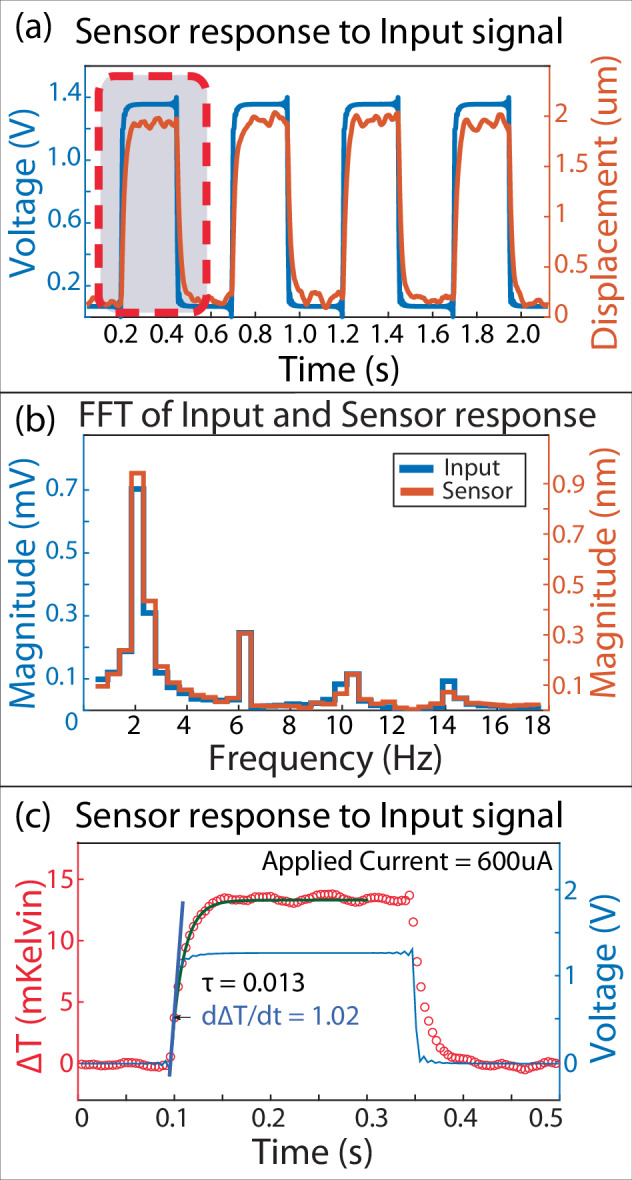
The step power response of the 1.0 mm × 1.0 mm membrane. **a** A 600 *μ*A current with 50% duty-cycle and 0.5-s pulses is applied to the micro-heater (Fig. 5). **b** The Fast Fourier transform (FFT) of both the input signal and the sensor response. The sensor picks up higher harmonic frequency components (ripples) from the applied power as it acts as a low-band pass filter. The steady state is fitted with the average value of these higher harmonic frequency components. The voltage across the micro-heater is 1.3 V. The step power to the micro-heater is *P*_heater_ = 0.78 mW. The heat power input to the air chamber is assumed to be half of the *P*_heater_, *P*_in_ = *P*_heater_/2 = 0.39 mW. The sensor response data, red dotted line in (**a**), is fitted with $\Delta T={P}_{{\rm{in}}}{R}_{{\rm{th}}}\cdot [1-\exp (-t/\tau )]$. From the curve fit time constant, *τ*, is 0.013 s, and the slope, Δ*T*/*dt*∣_*t*=0.1_, is 1.02, as shown in (**c**)

**Fig. 10:**
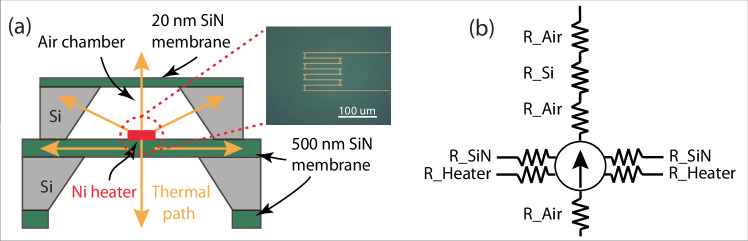
Thermal paths from the micro-heater. **a** Thermal paths of the applied heat from the Ni heater (100 × 100 *μ*m). The inset is the top view of the micro-heater. **b** Equivalent thermal resistance network of the device. ${R}_{{\rm{SiN}}}$ is calculated in cylindrical coordinates. *R*_Air-Si-Air_ and *R*_Air_ are calculated in spherical coordinates

**Fig. 11:**
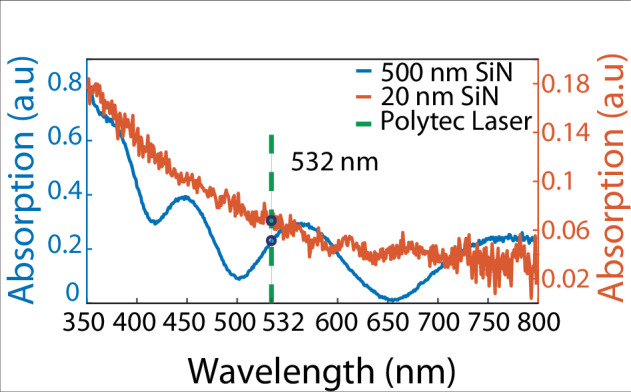
UV–VIS spectroscopy absorption data on 20 nm thick membrane and 500 nm thick SiN films

**Fig. 12:**
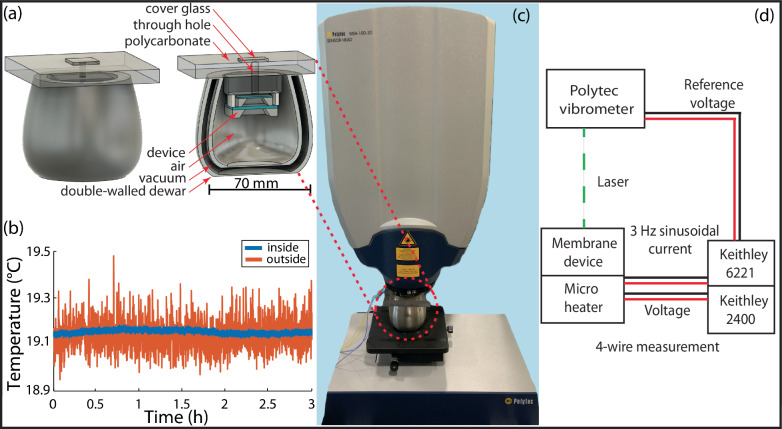
Measurement setup. **a** A 3-D rendering of the device placed inside the double-walled dewar. For the measurements of the membrane deflection, the device was placed inside this double-walled dewar (2.5” tall and 2.5” diameter), which served as an environmental chamber and reduced the effects of any abrupt temperature or pressure change. **b** The temperature comparison between the outside and inside environment using two PT-100 sensors for 3 h. **c** An optical image of the measurement setup, Polytec MSA-100, and the device placed inside the double-walled dewar. **d** A Keithley 6221 DC & AC current source and a Keithley 2400 connected to the micro-heater in a 4-wire measurement configuration. A 3 Hz sinusoidal current was applied to the heater from the Keithley 6221 DC & AC current source. The Keithley 2400 was used to simultaneously measure the voltage drop across the heater, which was also used as a reference voltage signal to the Polytec MSA-100-3D. The electrical current and the measured voltage were used to calculate the applied power to the micro-heater

**Fig. 13:**
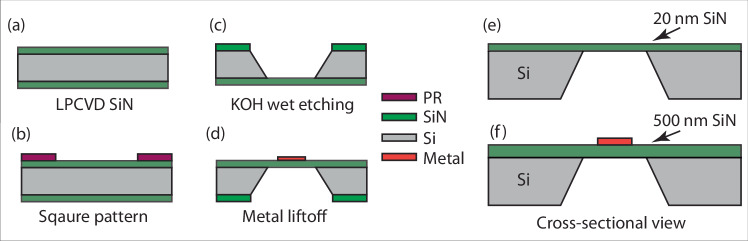
The fabrication process and cross-sectional view of the 20 nm thick SiN membrane and 500 nm thick SiN film. **a** A low-stress LPCVD SiN film deposition on a silicon wafer. **b** Square patterns patterned by optical lithography followed by SiN film etch by RIE. **c** Wet KOH of silicon. **d** Metal lift-off to integrate the micro-heater. **e** The cross-sectional view of the 20 nm thick SiN membrane device (no metal layer). **f** The cross-sectional view of the 500 nm thick SiN film with a metal micro-heater

## Data Availability

The data supporting the findings are displayed in the main text and the appendices. All raw data are available from the corresponding authors upon request.

## Appendices

### Stiffness measurement with a concentrated load

To measure and estimate the stiffness of the 20 nm thick membrane, a force curve method with a commercial AFM and an FEM (ANSYS) is used. A summary of the stiffness of the membranes from the concentrated force load is shown in Table 7.

**Table 7:** Membrane size, *k*_*a*,*c*_, *k*_*ω*,*c*_, *k*_*m*_, and *k*_A,F_ in N/m

Membrane size	*k*_*a*,*c*_	*k*_*ω*,*c*_	*k*_*m*_	*k*_*A*,*F*_
0.5 mm × 0.5 mm	0.67	0.70	0.96	0.42
1.0 mm × 1.0 mm	0.67	0.73	0.49	0.39
3.0 mm × 3.0 mm	0.67	0.78	0.61	0.36

As discussed in the section “Device configuration and the principle of operation”, the stiffness of the square membrane with a distributed load is four times larger than that with a concentrated load. Both *k*_*ω*_ and *k*_*A*,*ω*_ presented in the section “Results” are the stiffness with a distributed load. The analytically calculated stiffness from Eq. (6), which is for linear analysis, is an order of magnitude higher than the lowest values obtained by the nonlinear ANSYS force–distance analysis. The reason for the discrepancy between *k* values from Eq. (6) and the measured values is that Eq. (6) is for the small deflection (less than the plate thickness), while the measured values are from a large deflection (~1 *μ*m) of the membrane. The stiffness values from the ANSYS force–distance analysis are smaller than the measured values. Because the force is applied to a single node at the center of the membrane, the single node may be high, resulting in low stiffness.

In Table 7, *k*_*a*,*c*_ = *k*_*a*,*d*_/4 = 0.67 N/m and *k*_*ω*,*c*_ = *k*_*ω*,*d*_/4 are from ref. ^25^. *k*_*m*_ is calculated from measured force–distance slopes with an AFM, Eq. (IA.1). *k*_*A*,*F*_ is obtained from ANSYS force–distance analysis.

#### AFM force curve measurement

Using Hooke’s law, *F* = − *k**δ*, the membrane stiffness can be estimated by applying a known force at the center of the membrane and measuring the deflection. An AFM is a tool for applying a known force (sub-nN resolution) and measuring the deflection (0.1 nm resolution) of delicate micro/nanoscale samples^45–47^.

AFM force curve measurement has been used to estimate an unknown cantilever stiffness, *k*_*c*_^46^^,48^. The fundamental resonant frequency (59.5 kHz) and Q (148) of an AFM probe (AppNano FCLA-5) are obtained by a thermal tuning method with SOLVER NANO AFM from ND-MDT as shown in Fig. 6a. The Sader’s method^30,49^ along with a Lorentzian fit is used to get the stiffness of the probe, 1.36 N/m. This stiffness is suitable for the membrane stiffness measurement because the smallest value *k*_*m*_ of the membrane is measured to be ~ 0.5 N/m. *k*_*c*_ should be matched to *k*_*m*_ within a dynamic range such that 0.3 *k*_*m*_ < *k*_*c*_ < 3.0 *k*_*m*_^50^.

Once the AFM probe stiffness is known, the stiffness of the 20 nm thick membrane can be obtained in two steps^48^. The first step is to obtain a force vs. distance curve with the calibrated AFM probe pushed against a flat and hard surface, e.g., the frame (silicon) of the membrane. The slope, *S*_*c*_, is measured and used to calculate the membrane’s stiffness. The next step is to place the AFM probe on the center of the membrane and obtain the force–distance curve as shown in Fig. 6b. The *y*-axis is in nA (cantilever deflection, DFL, signal in photodetector current) instead of the force in N. The slope, *S*_*m*_, is the measure of the combined stiffness of the cantilever (AFM probe) and the membrane. The stiffness of the membrane can be calculated from

IA.1 $${k}_{m}={k}_{c}\frac{{S}_{m}\cos \theta }{{S}_{c}-{S}_{m}},$$

where *k*_*c*_ is the stiffness of the AFM probe and *θ* is the angle between the AFM probe and the membrane^46^^,48^. The slopes calculated in Fig. 6b are in nA/nm. The slope for the frame is *S*_*c*_ = 8.6 pA/nm, and that for the 1.0 mm × 1.0 mm membrane is *S*_*m*_ = 2.3 pA/nm. SOLVER NANO AFM is used to get force–distance curves, and the probe is mounted at 10^o^ angle with respect to the membrane surface. The uncertainty of this method is reported to be between 10% and 30%^50,51^. The stiffness of the membranes measured by a cantilever with a 1.36 N/m stiffness are summarized in Table 8.

**Table 8:** Membrane size, *S*_*m*_ (pA/nm), and *k*_*m*_ (N/m) measured by the AFM method with *k*_*c*_ = 1.36 N/m and *S*_*c*_ = 8.6 (pA/nm)

Membrane size	*S*_*m*_	*k*_*m*_
0.5 mm × 0.5 mm	2.9	0.68
1.0 mm × 1.0 mm	2.3	0.49
3.0 mm × 3.0 mm	2.7	0.61

#### Force curve measurement: ANSYS

A static analysis for the force–distance curve is performed using the same element (SHELL281), boundary conditions, and prestress. Forces from 0 N to 200 nN with 20 nN increments are applied to a node at the center of the membrane, and the displacements at each force are determined. The slope of the force–distance curve is the stiffness of the membrane, *k*_*A,F*_. The deformed membrane and the force–distance curves are shown in Fig. 7. The force–distance curve is non-linear at larger forces (>80 nN). Therefore, further development of the physical contact model between the probe with a finite curvature at the tip and the membrane is required to calculate the stiffness better. The stiffness’s average value is compared with the stiffness obtained by other methods. *k*_*A,ω*_ and *k*_*A,F*_ are summarized in Table 9.

**Table 9:** Membrane size, *k*_A,*ω*_ (N/m), and *k*_A,*F*_ (N/m) from the ANSYS analysis

Membrane size	*k*_A,*ω*_	*k*_A,*F*_
0.5 mm × 0.5 mm	2.71	0.42
1.0 mm × 1.0 mm	2.70	0.39
3.0 mm × 3.0 mm	2.89	0.36

### Frame modes

The mode shape at 11 kHz scanned using the Polytec MSA-100-3D is shown in this appendix. The mode shape at 11 kHz of the membrane with an amplitude compatible with the fundamental frequency, *f*_1,1_, is shown in Fig. 8a. The mode of 11 kHz is generated due to the substrate and the membrane coupling. A scanned image of the frame alone (without the membrane) is shown in Fig. 8b, in which a vibration mode of the frame with a large deflection is evident. From this analysis, we conclude that the pronounced peak in the membrane response at 11 kHz is mainly caused by the vibration response of the membrane frame.

### Device’s thermal parameters

Figure 9a shows the sensor’s response to the applied step power on the 1.0 mm × 1.0 mm membrane. The FFT results of the sensor response and the 2 Hz input signal are shown in Fig. 9b. The results reveal additional higher harmonic frequency components (ripples) from the input signal that are picked up by the device as it acts as a low-band pass filter. For calculating the device’s thermal parameters, such as the time constant, thermal resistance, and thermal capacitance, the steady state was fitted with the average value of these higher harmonic frequency components shown in Fig. 9c. The thermal parameters can be extracted using $\Delta \,T\,=\,{P}_{{\rm{in}}}{R}_{{\rm{th}}}\,\cdot \,[1\,-\,\exp (-t/\tau )]$. A step power (0.39 mW of Joule heating) at the time *t* = *t*_0_ is applied to the micro-heater shown in Fig. 5. The temperature change converted from the deflection of the 20 nm thick membrane using Eq. (7) is shown in Fig. 9c.

The time constant of the device can be written as

IC.2 $$\tau ={R}_{{\rm{th}}}\cdot {C}_{{\rm{th}}}$$

where *R*_th_ is the thermal resistance of the device, and *C*_th_ is the thermal capacitance.

The thermal resistance can be written as

IC.3 $${R}_{{\rm{th}}}=\frac{\Delta {T}_{{\rm{ss}}}}{{P}_{{\rm{in}}}},$$

where Δ*T*_ss_ is the steady-state temperature rise and *P*_in_ is the power input.

The thermal capacitance can be written as

IC.4 $${C}_{{\rm{th}}}={P}_{{\rm{in}}}\cdot {\left({\left.\frac{d\Delta T}{dt}\right\vert }_{t = {t}_{0}}\right)}^{-1},$$

where $d\Delta T/dt{| }_{t = {t}_{0}}$ is the slope of the step response at *t* = *t*_0_, where *t*_0_ is the time the step power input is applied. The thermal parameters of the 1.0 mm × 1.0 mm square membrane device are *τ* = 13 × 10^−3^ s, *f*_*c*_ = 1/(2*π**τ*_air_) ~ 12 Hz, *G*_th_ = 1/*R*_th_ = 28 × 10^−3^ W/K.

The measured *G*_th_ is compared to the calculated *G*_th_ by using the Fourier heat equation, ${\dot{Q}}_{{\rm{res}}}={G}_{{\rm{th}}}\times \Delta {T}_{{\rm{res}}}$, where the ${\dot{Q}}_{{\rm{res}}}$ is the applied power to the device. From the plate theory, the temperature resolution for the plate approach is given by Δ*T* = *α* ⋅ (*D* ⋅ *δ*/*ρ**R*_*s*_*a*^4^), where *α* is 1/0.00126, *a* is half of the square membrane’s length, *D* is the flexural rigidity, *ρ* is the density of the gas, and *R*_*s*_ is the mass-specific gas constant^25,27^. Using *δ* = 0.74 nm, the corresponding Δ*T* is 5.60 × 10^−9^ K. The *G*_th_ is 37.5 × 10^−3^ W/K. The membrane approach to get the thermal conductance can be used with the deflection from the step power response measurement, where the deflection is larger than the thickness of the membrane. The deflection, *δ*, is 1.89 × 10^−6^ m. Δ*T* using Eq. (7) is 1.44 × 10^−3^ K. The *G*_th_ = 61.4 × 10^−3^ W/K. The calculated *G*_th_ from the plate approach and the membrane approach agrees with the measured *G*_th_.

Figure 10 shows (a) the thermal path of the applied heat and (b) the equivalent thermal resistance network. To compare the measured and calculated (the Fourier heat equation) thermal conductance with the thermal conductance of the device, the lumped thermal resistance of the device was calculated from the resistance network as shown in Fig. 10b. The thermal resistance of each component of the device is considered. These are the resistances of the Ni heater (*R*_Ni_), the 500 *μ*m SiN film (${R}_{{\rm{SiN}}}$), the air chamber (*R*_air_), and the Si (*R*_Si_). The thermal resistance of the air chamber (*R*_air_) and the Si (*R*_Si_) are in series, and the thermal resistance of the Ni heater (*R*_Ni_) is parallel to that of the SiN (${R}_{{\rm{SiN}}}$). The total lumped thermal resistance of the device is ${R}_{{\rm{total}}}^{-1}={R}_{{\rm{Ni}}}^{-1}+{R}_{{\rm{SiN}}}^{-1}+{R}_{{\rm{air}}}^{-1}+{R}_{{\rm{Si}}}^{-1}+{R}_{{\rm{others}}}^{-1}$. *R*_others_ includes the thermal resistance from convection and radiation. *R*_Ni_, ${R}_{{\rm{SiN}}}$, and *R*_Air-Si-Air_ are calculated in Cartesian, cylindrical, and spherical coordinates, respectively, Table 10. The thermal resistance of each element is summarized in Table 10. The total lumped thermal resistance, *R*_total_ = 39.21 K/W. ${G}_{{\rm{SiN}}}=1/{R}_{{\rm{SiN}}}$ dominates the total lumped thermal resistance.

**Table 10:** Thermal resistance of each component of the device

	Ni heater	SiN film	Air chamber (Air-Si-Air)
Equation	*l*_h_/*k*_Ni_*t*_h_*w*_h_	$\ln \left({r}_{o}/{r}_{i}\right)/2\pi {t}_{SiN}k$	$4\pi k\left({r}_{o}{r}_{i}\right)/\left({r}_{o}-{r}_{i}\right)$
Thermal conductivity (*k*) [W/mK]	91	30	*k*_Air_ = 0.0262, *k*_Si_ = 148
Thickness (*t*) [m]	80 × 10^−9^	500 × 10^−6^	N.A.
Effective radius (*r*) [m]	*l*_h_	$a/\sqrt{\pi }$	$a/2\sqrt{\pi }$
Thermal resistance (*R*_th_) [K/W]	~4.5 × 10^11^	~39.14	~4.9 × 10^4^

### Absorption of the laser in SiN films

The wavelength and the laser power of the Polytec MSA-100-3D are 532 nm and 14 *μ*W, respectively. The absorption using UV-VIS Spectroscopy (Agilent Carry 5000 UV-VIS-NIR) of both the 20 nm thick membrane and 500 nm thick SiN films is shown in Fig. 11. Radiation pressure from the Polytec laser on the 20 nm membrane was calculated using the Poynting vector^52^. From Eq. (IE.5), *P*_net_ = 2*I*_*f*_/*c* where *I*_*f*_ is the incident power of the laser and *c* is the speed of light. Based on Fig. 11, about 7% of the total incident light was used for *I*_*f*_. The calculated radiation pressure from the reflected laser on the 20 nm membrane was 260 *μ*Pa. The corresponding temperature change, from Eq. (2), was 0.7 *μ*K. The calculated radiation pressure from the Polytec laser adds to the DC offset as discussed in the section “Results”. Although the Polytec laser intensity fluctuation data was unavailable, an estimation of 10% (0.07 *μ*Pa) shows it is a small portion of the measured NET. The majority of measured NET is from the environmental noise, the photodetector noise, and equipment noise, as discussed in the section “The noise equivalent of temperature”.

### Radiation pressure from the Polytec laser

For all of the measurements of the membrane deflection, the device was placed inside a cylindrical double-walled dewar (2.5” tall and 2.5” diameter), which served as an environmental chamber. A 0.5” thick transparent polycarbonate plate was placed on top of the dewar to isolate the device from the effect of the outside temperature drift, as shown in Fig. 12a. The Polytec MSA-100-3D laser was focused(~4 *μ*m diameter) on the 20 nm thick membrane and reflected back through the transparent polycarbonate. The wavelength and the laser power of the Polytec MSA-100-3D were 530 nm and 14 *μ*W, respectively. To estimate the absorption of the laser power on the device, the absorption rates of the laser on the 20 nm thick membrane and 500 nm thick SiN film were measured as described in the appendix “Absorption of the laser in SiN films”. The absorption of the laser on the 20 nm thick SiN membrane was negligible and on the 500 nm thick SiN film was less than 10% of the power of the incident beam. Radiation pressure (*P*_net_) from the reflected Polytec laser on the 20 nm thick membrane was calculated using the Poynting vector^52^. From Eq. (IE.5)

IE.5 $${P}_{{\rm{net}}}=2\frac{{I}_{f}}{c},$$

where *I*_*f*_ is the incident power of the laser and *c* is the speed of light. Based on Fig. 11, about 7% of the total incident light was used for *I*_*f*_. The calculated radiation pressure from the reflected laser on the 20 nm membrane was 260 *μ*Pa. This calculated radiation pressure adds to the DC offset. The laser power absorbed on the SiN film may continuously heat the air chamber, resulting in a deflection of the 20 nm thick membrane. This deflection can be considered an addition to the existing DC offset resulting from the radiation pressure and the temperature difference between the sealed air chamber and the external environment. By modulating the sources and referencing the modulation frequency to the Polytec MSA-100-3D controller (phase-sensitive detection technique), we could minimize the membrane deflection caused by the environmental temperature difference.

### Measurement setup

A 3-D rendered image of the device inside the double-walled dewar is shown in Fig. 12a. For the measurements of the membrane deflection, the device was placed inside this cylindrical double-walled dewar (2.5” tall and 2.5” diameter). This double-walled dewar was filled with air and served as an environmental chamber for the device. A 0.5” thick transparent polycarbonate plate was placed on top of the dewar with a hole for the Polytec laser access. A cover glass was placed to seal the hole on the polycarbonate plate. The polycarbonate plate was sealed to the dewar using silicone grease to further isolate the device from outside environmental effects, such as any abrupt temperature or pressure change that could affect the measurement. Figure 12b shows the temperature comparison between the outside and inside environment for 3 h. The temperature data shown in Fig. 12b was collected using two PT-100 sensors. An optical image of the device under Polytec MSA-100-3D is shown in Fig. 12c. Figure 12d shows a Keithley 6221 DC & AC current source and a Keithley 2400 connected to the micro-heater in a 4-wire measurement configuration. A 3 Hz sinusoidal current was applied to the heater from the Keithley 6221 DC & AC current source. The Keithley 2400 was used to simultaneously measure the voltage drop across the heater, which was also used as a reference voltage signal to the Polytec MSA-100-3D. The electrical current and the measured voltage were used to calculate the applied power to the micro-heater.

### Device fabrication process

The device used in this work consists of a 20 nm thick SiN membrane and a 500 nm thick SiN film forming an air chamber. Fabrication of both membranes follows the same process. Figure 13 shows the fabrication process flow: (a) A low-stress LPCVD SiN film is deposited on a silicon wafer by Rogue Valley Microdevices. (b) Square patterns are patterned on the wafer by optical lithography and the SiN film is etched by RIE. (c) The exposed silicon is etched in a wet potassium hydroxide solution (KOH, 30% w/w) at 60 °C for 28 h. (d) Metal lift-off to integrate the micro-heater. (e) The cross-sectional view of the 20 nm thick SiN membrane device (no metal layer). (f) The cross-sectional view of the 500 nm thick SiN film with a metal micro-heater.
